# The Gas- and Condensed-Phase Efficacy of Functionalized Phosphorus Flame Retardants for Cotton Fabric: Phenyl vs. Phenoxy Groups

**DOI:** 10.3390/polym17070924

**Published:** 2025-03-28

**Authors:** Raphael Otto, Ava Cardona, Alexander M. Preußner, Wael Ali, Jochen S. Gutmann, Thomas Mayer-Gall

**Affiliations:** 1 Institute of Physical Chemistry and Center for Nanointegration (CENIDE), University of Duisburg-Essen, Universitätsstraße 2, 45117 Essen, Germany; ali@dtnw.de (W.A.); jochen.gutmann@uni-due.de (J.S.G.); 2 Deutsches Textilforschungszentrum Nord-West gGmbH, Adlerstr 1, 47798 Krefeld, Germany

**Keywords:** flame retardancy, cotton fabric, phosphorus flame retardants, condensed-phase mechanism, phenyl, phenoxy

## Abstract

This study explores how functionalized aromatic P-FRs, specifically phenyl- and phenoxy-based phosphoric acid derivatives, influence the flame retardancy of cotton textiles. By systematically investigating derivatives with varying degrees of phenyl, phenoxy, and acidic hydroxyl terminations, alongside ortho-phosphoric acid as a reference, this work aimed to elucidate the role of aromaticity and functional group composition on both gas- and condensed-phase flame retardant efficacy. Cotton fabrics were treated with comparable phosphorus loadings (~3 g/m^2^), quantified using inductively coupled plasma optical emission spectroscopy (ICP-OES), to evaluate the gas- and condensed-phase efficacy of the flame retardants. Notably, derivatives with a higher number of acidic hydroxyl terminations exhibited the best flame retardant performance, enhancing char formation through dehydration and condensation reactions during combustion. Thermal analysis (TGA) and microscale combustion calorimetry (MCC) confirmed that phenoxy systems catalyze cotton decomposition more effectively, promoting dehydration through the hydrolysis of phenoxy groups. Furthermore, IR analysis of evolved gases revealed a significant reduction in volatile emissions for phenoxy systems, while this was not observed for phenyl derivatives. These findings underscore the importance of robust condensed-phase mechanisms for achieving effective flame retardancy in cotton textiles.

## 1. Introduction

Cotton, a widely used natural fiber known for its excellent breathability, comfort, and versatility, is highly flammable, posing serious fire risks in applications ranging from apparel to home textiles [1]. To mitigate this risk, flame retardant (FR) finishing coatings are applied to cotton to enhance its thermal stability and fire resistance. Among the different FR classes, phosphorus-based flame retardants have garnered considerable attention for their efficiency, environmental compatibility, and versatility. These compounds have especially been in the spotlight as an alternative for hazardous and environmentally unfriendly halogenated FRs, which are increasingly becoming prohibited due to stricter governmental regulations [2,3].

Phosphorus-based flame retardants greatly promote char formation, inhibit pyrolysis, and suppress flame propagation. The mode of action is highly influenced by the chemical environment of the phosphorus center as heteroatoms like nitrogen and oxygen increase the acidity and the char formation properties, while P-C bonds are pyrolyzable and are believed to form POx radicals that show high activity in the gas phase [2]. Phosphorus is often used in combination with synergistic elements, most notably nitrogen (found in species like phosphazenes [4,5,6] and phosphoramidates [3,7,8,9]) and sulfur [10,11,12], or beneficial materials like silica [13,14,15,16], which enhance the effectiveness of the FR and permit smaller add-on values, increasing the economic feasibility.

In recent years, there has been growing interest in aromatic phosphorus-based FRs for cotton fabric, with a particular focus on DOPO (9,10-dihydro-9-oxa-10-phosphaphenanthrene-10-oxide)-based derivatives [17,18,19,20,21] and phenyl/phenoxy-functionalized species [22,23,24,25]. In comparison to ethoxy/methoxy–phosphorus ester derivatives, the FR efficacy of aromatic functionalized P-based compounds is limited, as the bulky substituents decrease the interaction with the cellulosic substrate [9]. Phenyl-functionalized phosphorus compounds, however, offer distinct advantages, for instance, as adjuvants in superhydrophobic coatings [26,27]. Increasing the hydrophobicity of the fabric is vital for many applications, as it increases the washability, water repellency, and self-cleaning properties [27]. In particular, the washability remains an important bottleneck for the development of potent FRs for textiles, rendering aromatic functionalized species promising, despite their lower flame retardant efficacy compared to alkoxy and acid derivatives [28]. Prominent strategies to enhance the washing fastness of cotton fabric encompass the sol-gel approach of functionalized silane precursors [11,13,14,17], and the grafting of phosphoric acid derivatives via dicyandiamide (DCD) and urea treatments [29,30,31].

Despite their promising characteristics, the mechanisms by which phenyl-functionalized phosphorus FRs interact with cotton during thermal decomposition remain poorly understood. Similarly, there is a gap in fundamental studies on the influence of different oxidation states and chemical surroundings on the FR mechanism. Few systematic studies on aromatic-based P-FRs have been performed for epoxy resins and composites, assessing either phosphates or phosphine oxides to be more effective depending on the matrix, with phosphates portraying a higher activity in the condensed phase [32,33,34,35]. Recently, Qin et al. have been analyzing the impact of the oxidation state of aromatic P-based compounds on the degradation pathway in a theoretical study [36]. It was proposed that increasing the oxidation state of the phosphorus center promotes cleavage of P-C bonds, while the favorable dissociation site is located at the P-O bond for phenoxy systems. However, in their study, the interaction between the polymer substrate and the FR was not investigated, which is a significant simplification, particularly for reactive substrates like cellulose.

To investigate their hypothesis experimentally, we employed a comparative analysis between phenyl- and phenoxy-functionalized phosphoric acid derivatives as finishing coatings for cotton fabric. For this, we consecutively substituted a phenyl/phenoxy group with an OH group (0 > 3), increasing the interaction with the cellulose substrate gradually and determining the degree of condensed phase action as a function of OH groups, while maintaining comparable P loadings of ~3 g/m^2^. This comparison enabled us to correlate differences in the mode of action directly to the underlying chemical structure. It was decided to employ phosphoric acid (H_3_PO_4_) derivatives because acidic OH groups function as binding sites for the substrate and, while they are beneficial for substrate interaction, they can negatively impact the washing resistance, a challenge frequently addressed in the literature [23,26,37,38].

The successful finishing of samples was confirmed using FT-IR, inductively coupled plasma optical emission spectrometry (ICP-OES), and scanning electron microscopy (SEM). The flame retardant efficacy was determined using the standardized flame test according to EN ISO 15025:2016 [39], while the combustion behavior was assessed using micro-scale combustion calorimetry (MCC) and the thermal behavior was assessed using thermogravimetric analysis (TGA). Evolved gases were analyzed using IR spectroscopy (TG-IR), the phosphorus content in the residual char was determined using ICP-OES, and the graphitization degree was assessed using Raman spectroscopy.

## 2. Materials and Methods

### 2.1. Materials

Commercial woven cotton fabric (CO, plain weave, 170 g/m^2^) was supplied by wfk Testgewebe GmbH, Brüggen, Germany. Triphenyl phosphorus oxide (TPPOX, 98%) was purchased from Sigma-Aldrich, St. Louis, MO, USA. Phenyl phosphate (PP, 99%) was purchased from TCI chemicals (Eschborn, Germany). Diphenyl phosphate (DPPOH, 97%), Diphenylphosphinic acid (DPPA, 99%), Phenylphosphonic acid (BPA, 98%), and Triphenyl phosphate (TPP, 98%) were purchased from abcr GmbH (Karlsruhe, Germany).

Tetrahydrofuran (THF, 99.9%) was purchased from Fisher Scientific GmbH (Schwerte, Germany). Ethanol (96%) and ortho-phosphoric acid (H_3_PO_4_, 85%) were purchased from Carl Roth Gmbh (Karlsruhe, Germany).

### 2.2. Coating Procedure

Pristine cotton fabric was coated via pad-dry technique. For that, an appropriate amount of FR was dissolved in either ethanol or THF to achieve a concentration that led to approximately 3 g/m^2^ phosphorus on the fabric. Depending on the compound, the solution was poured into a tray ^[t]^ and a 16 × 10 cm^2^ square textile piece was immersed in the solution shortly from both sides, while other solutions were applied to the fabric via pipette ^[p]^. In the case of tray finishing, the coated textile pieces were padded with the LP Laboratory Padder from LAB-PRO GmbH (Wikon, Switzerland) and cured in a laboratory oven at 80 °C for 30 min.

The pipetting procedure was as follows: 4 mL of FR-solution was applied to the fabric twice (8 mL in total), using an Eppendorf pipette, followed by padding. The procedure was repeated once, and the sample was subsequently cured as reported above. The process was repeated a variable number of times until the desired P add-on was reached, as indicated in Table 1. The weight gain after coating was also monitored and calculated according to Equation (1).

(1) $$weight-gain\left(\%\right)=\frac{\left[m_{1}-m_{0}\right]}{m_{1}}\times 100$$

where ($m_{0}$) and ($m_{1}$) are the average weight of cotton samples before and after treatment, respectively.

### 2.3. Vertical Flame Test

Vertical flame tests were conducted according to EN ISO 15025:2016 (Protective clothing—Protection against heat and flame—Method of test for limited flame spread, 10 s ignition) utilizing the Gester flammability tester (Model GT-C35B, Quanzhou, China). The tests implemented the surface ignition procedure, with a flame height set at 20 mm. Compliance with passing requirements was evaluated based on ISO 11611:2024 [40] standards. Test results are presented in Table 2. Char yield was determined by weighing circular cut-outs of unburned and burned specimen with a diameter of 12 mm. Videos of the flame tests and photographs of the specimen are provided in the ESI or can be downloaded online.

### 2.4. Spectroscopy

#### 2.4.1. FT-IR

Fourier transform infrared spectroscopy (FTIR) measurements were performed using an IR Prestige-21 instrument (Shimadzu Deutschland GmbH, Duisburg, Germany) in attenuated total reflection (ATR) mode. Each sample was analyzed with an average of 40 scans at a resolution of 4 cm^−1^. The ATR setup featured a golden gate equipped with a diamond crystal (Specac Ltd., Orpington, UK). Finishing of cotton samples was confirmed by subtracting a normalized reference spectrum of pristine cotton from the normalized sample spectrum, according to Equation (2).

(2) $$\Delta Abs.=\frac{{Abs.}_{Sample}}{{\max(Abs.}_{Sample})}-\frac{{Abs.}_{Cotton}}{{\max(Abs.}_{Cotton})}$$

with Abs. = IR-Absorbance.

#### 2.4.2. Raman Spectroscopy

Raman spectroscopy was conducted using an EZRaman-N (Enwave Optronics, Inc., Irvine, CA, USA) with an excitation wavelength of 532 nm, spectral resolution of 7 cm^−1^, and an integration time of 20 s. The original spectra are found in the ESI (Figure S3).

### 2.5. TGA

Thermogravimetric analyses (TGA) were performed using a Discovery TGA 55 instrument (TA Instruments, Hüllhorst, Germany) over a temperature range of 100–800 °C. Approximately 10 mg of the sample was placed in an alumina crucible (Al_2_O_3_). The temperature was initially maintained at 100 °C for 5 min to remove adsorbed moisture, followed by heating at a rate of 20 K/min under a nitrogen atmosphere with a gas flow rate of 90 mL/min.

### 2.6. TG-IR

Evolved gaseous products were analyzed by coupling the same TGA instrument with the previously mentioned IR spectrometer (TG-IR, 4 scans, res. 4 cm^−1^, 600–4000 cm^−1^) using a gas cell and a heated stainless teel transfer line maintained at 240 °C.

### 2.7. MCC

Micro-scale combustion calorimeter (MCC) measurements were conducted using a Fire Testing Technology Ltd. instrument (East Grinstead, UK), following ASTM D 7309 [41] Method A. During each experiment, the sample was pyrolyzed up to 750 °C at a heating rate of 1 K/s under a nitrogen flow of 80 mL/min. The resulting volatile degradation products were then combusted at 900 °C in a combustion chamber, where they were mixed with pure O_2_ supplied at a flow rate of 20 mL/min. The data collected were analyzed using MATLAB 2024b software (MathWorks, Natick, MA, USA). Each sample was tested in triplicate, and the reported results represent the average of these measurements.

The fire growth capacity (FGC) was calculated using Equation (3):

(3) $$FGC=\left(\frac{THR}{T_{95\%}-T_{5\%}}\right)\left(\frac{T_{95\%}-298\;K}{T_{5\%}-298\;K}\right)$$

where THR represents the total heat release, while T_5%_ and T_95%_ refer to the temperatures corresponding to the ignition and burnout points, respectively. These temperatures are determined from the 5% and 95% points of the integrated heat release rate (HRR) curve.

### 2.8. SEM

Scanning electron microscopy (SEM) was performed using a Hitachi S-3400 N II SEM instrument (Hitachi HighTech Europe GmbH, Mannheim, Germany) operating at an accelerating voltage of 10 kV. To improve conductivity and imaging quality, the sample surfaces were coated with gold using a Quorum Emitech K500X sputter coater (Ashford, Kent, UK) under vacuum for 4 min.

### 2.9. ICP-OES

ICP-OES analysis was conducted to determine the phosphorus content using a Varian 720-OES instrument (Varian Inc., Darmstadt, Germany) in triplicate. Circular samples with a diameter of 12 mm were digested in 8 mL of 69% HNO_3_ solution using a microwave-assisted digestion process for 1 h with a Mars Xpress instrument (CEM GmbH, Kamp-Lintfort, Germany). The resulting solution was then diluted with 17 mL of Millipore water, and any residues were removed using a filter cannula. The phosphorus content was quantified both as a percentage and relative to the surface area (area density of phosphorus, *aₚ*) using Equation (4):

(4) $$a_{P}\;(\frac{g}{m^{2}})=\frac{c_{ICP}V}{a}$$

where c*_ICP_* represents the concentration measured using ICP-OES, with a volume (V) of 25 mL. The phosphorus content and area density of charred samples were determined using circular sections taken after the vertical flame test.

The percentage mass loss of phosphorus (Δa_p_) before and after burning was calculated using Equation (5):

(5) $$\Delta a_{P}=\frac{a_{P,Char}\;\left(\frac{g}{m^{2}}\right)}{a_{P}\;(\frac{g}{m^{2}})}\times 100\%$$

## 3. Results

Aromatic functionalized phosphoric acid-based FRs are frequently utilized to introduce flame retardancy in complex hydrophobic coatings. Both phenyl [23,26,27,37] and phenoxy [22,23,24] groups are readily utilized for cotton fabric, with their proposed FR mechanism and efficacy described interchangeably. Both material classes show activity in the gas and condensed phase, as phosphoric acid derivatives tend to oligomerize and polymerize to pyrophosphates and polyphosphoric acid, and catalyze the dehydration of cellulose. Meanwhile, aromatic-based compounds tend to evaporate and degrade via pyrolysis, generating PO radicals [1,2]. Despite extensive studies on phenyl- and phenoxy-functionalized FRs, to our knowledge, a direct comparison between these groups has not been conducted. As a result, the underlying flame retardant mechanisms of these compounds remain unclear, especially regarding their condensed- and gas-phase activities and their relative efficacy on a cotton substrate. Determining the intrinsic FR properties of both material classes will enable the design of more effective FR hybrid coatings in the future, providing clearer guidance in selecting between phenyl- and phenoxy-functionalized FRs.

**Figure 1:**
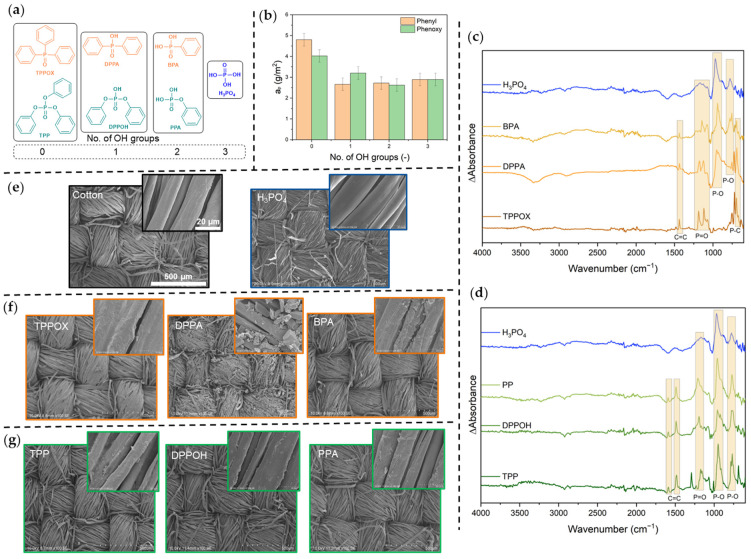
Phosphorus-based flame retardant (P-FR) compounds used in this study (**a**) and their corresponding area density of phosphorus on cotton fabric according to ICP-OES (**b**). Spectral change in IR absorbance after coating with phenyl (**c**) and phenoxy (**d**) derivatives. SEM images of pristine cotton, phosphoric acid (**e**), and phenyl- (**f**) and phenoxy-based coatings (**g**). The scales are identical for all images.

In this work, phenyl (triphenyl phosphorus oxide, TPPOX; diphenylphosphinic acid, DPPA; phenylphosphonic acid, BPA) and phenoxy phosphoric acid derivatives (triphenyl phosphate, TPP; diphenyl phosphate, DPPOH; phenyl phosphate, PPA) were applied to cotton fabric and analyzed regarding their FR efficacy and mechanism (Figure 1a). First, the surface morphology and composition will be discussed, followed by the thermal and calorimetric characteristics. Subsequently, the FR efficacy will be identified and the action in both the condensed and gas phase will be illuminated by analyzing the residual char and emitted volatiles. Afterwards, our findings will be compiled to propose an appropriate FR mechanism and evaluate the suitability of phenyl and phenoxy moieties for flame retardant purposes.

### 3.1. Characterization and Coating Procedure

FR compounds were applied to cotton fabric via the pad-dry technique. For this, the cotton sample was either immersed in an FR-containing solution or the solution was applied to the fabric with a pipette, as it was challenging to reach similar area densities of phosphorus values (~3 g/m^2^, Figure 1b). This value was selected because it represents the lowest possible add-on for the benchmark molecule H_3_PO_4_ required to exhibit self-extinguishing behavior in the flame test. The hydrophobic compounds (less than two OH groups) tended to crystallize on the semicrystalline surface and form deposits, while add-on values higher than 3 g/m^2^ were obtained with the hydrophilic compounds (with at least two OH groups) when immersed in a tray. With the pipette method, both higher uniformity and smaller add-ons were achieved, as the volume and the concentration of the solution could be controlled directly and evenly applied to the specimen. It was decided not to adjust the phosphorus loadings of TPP and TPPOX, as their observed FR efficacy remained comparatively poor even at significantly higher loadings.

The surface composition was analyzed using IR spectroscopy (Figure 1c,d). For this, a reference spectrum of the cotton substrate was subtracted from the spectra of a coated specimen (ΔAbsorbance, Equation (1)) so that solely FR-originating signals remained. Intensive signals for aromatic C = C (1490/1440 cm^−1^ and 767 cm^−1^), P = O (~1200 cm^−1^), P-O (960 cm^−1^ and 767 cm^−1^), and P-C-bonds (700 cm^−1^) in the case of phenyl compounds were identified, confirming finishing of individual FR species.

The SEM images reveal thick FR coatings for both phenyl and phenoxy systems (Figure 1e–g), while the treatment with H_3_PO_4_ did not lead to perceivable morphological changes. The surface appears to be more porous and uneven in comparison to pristine cotton, as many of the FR materials solidified on top of and between the fibers. This phenomenon is more obvious for phenyl derivatives. The TPPOX sample, for instance, portrays apparently glued fibers, because of crystallization on the front of the surface, rather than between the fiber structure. The DPPA even shows crystallites and intensive porosity. This highlights the importance of incorporating DPPA into a multi-component finishing formulation, as the compound alone shows poor adhesion to the cotton substrate [26].

### 3.2. Thermal Behavior

**Figure 2:**
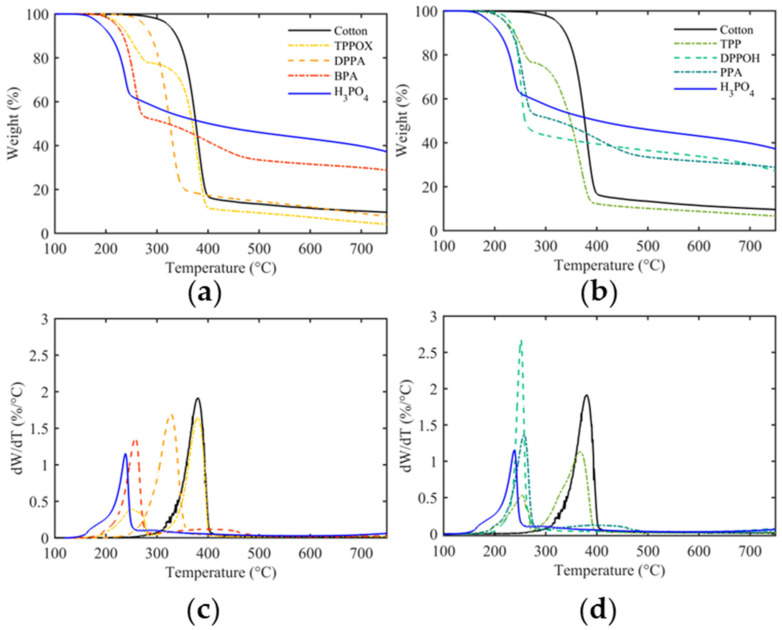
TG spectra of phenyl- (**a**) and phenoxy-functionalized P-FR coatings (**b**) and their derived DTG spectra (**c**,**d**).

The thermal properties of pristine and FR-finished cotton were evaluated using TGA, as shown in Figure 2. The temperatures at 5% (T_5%_) and maximum mass loss (T_max_), as well as the residue at T_max_ and at 700 °C, are presented in Table 3.

Under inert conditions, cotton undergoes pyrolysis, beginning at 324 °C (T_5%_) and reaching the maximum degradation rate at 380 °C (T_max_). As temperatures exceed 400 °C, the remaining residues are pyrolyzed further, leading to aromatic char, accompanied by the release of CO and CO_2_, leaving a residue of 10.2% at 700 °C [42]. The decomposition process is governed by two competing degradation mechanisms, either through the depolymerization of the glycosidic unit, or through the dehydration of cellulose. Generally, dehydration is preferable, since it leads to aliphatic char which acts as a protective layer, while depolymerization favors the formation of volatile products and tar, mainly levoglucosan. Depolymerization products act as fuel for the fire cycle and propagate thermal degradation once initiated [1].

When TPP and TPPOX were applied to the cotton fabric, no influence on the substrate’s degradation behavior could be observed, as evidenced by there being no changes in the T_max_ and a decrease in the residual char (7.4% and 5.0%, respectively). The reduction in residual char indicates that these compounds do not enhance char formation, which is typically associated with condensed-phase mechanisms. However, the unchanged T_max_ and the sharp DTG peak at T_max_, along with the rapid mass loss, indicate cellulose depolymerization as the dominant mechanism. This results in significant volatilization, implying that the gas-phase mechanism of these compounds is likely inactive for the cotton substrate. Both compounds decompose and evaporate in the N_2_ stream at 221 and 227 °C, suggesting weak adhesive interactions with the substrate.

**Figure 3:**
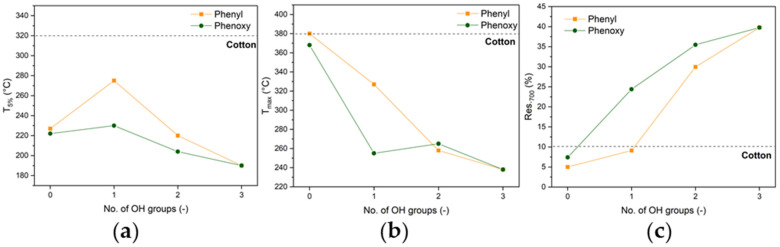
T_5%_ (**a**) and T_max_ temperatures (**b**), and the residue at 700 °C (**c**) are presented as a function of OH groups. The dashed line indicates the value of pristine cotton as a reference.

Increasing the number of OH groups changes the degradation pathway towards a two-step decomposition mechanism, with the first degradation regime starting at 190 °C, which is attributed to catalyzed cotton dehydration, and the second regime at T_max_ being ascribed to cotton depolymerization [43]. As a result, both T_5%_ and T_max_ decrease gradually, and are accompanied with a significant increase in charring (up to 39.8% for H_3_PO_4_). While the phenoxy derivatives produce comparably more char (Figure 3c) and accelerate cotton degradation stronger (Figure 3a,b), phenyl systems show more significant changes in T_5%,_ T_max_, Res._700_, and the number of OH groups (Figure 3). This indicates that improved thermal behavior is primarily influenced by the number of OH groups, while the phenyl moiety appears to have little to no impact. The trend is less obvious for phenoxy groups, as substituting a single phenoxy group leads to a strong mass loss at 255 °C, while substituting two phenoxy groups results in a DTG spectrum similar to that of phosphoric acid, but shifted to a higher T_max_. The sharp degradation profile observed for phenoxy-functionalized compounds might be the result of a dual-phase mechanism in which the FR simultaneously catalyzes cotton dehydration and volatilizes, leading to a sudden mass loss. Based on this behavior, we speculate that the degradation mechanism of phenoxy groups is increasingly changed from gas- to condensed-phase, possibly due to the hydrolysis of the phosphorus center, promoting char stabilization.

### 3.3. Combustion Behavior

**Figure 4:**
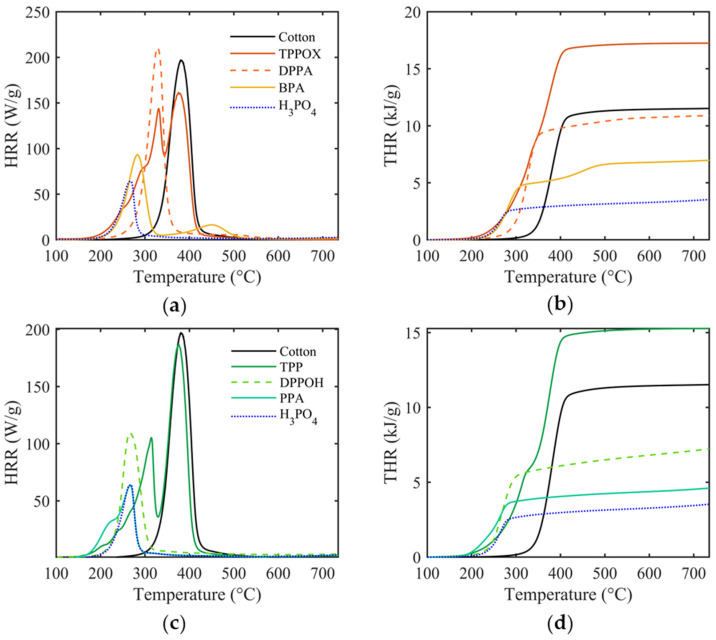
HRR and THR as a function of temperature for phenyl (**a**,**b**) and phenoxy derivatives (**c**,**d**).

Micro-combustion calorimetry (MCC) was utilized to characterize the combustion of emitted volatile species. Figure 4 presents the obtained heat release rate (HRR) and total heat release (THR) profiles as a function of temperature. The key combustion parameters, including fire growth capacity (FGC), peak heat release rate (pHRR), THR, and temperature at peak heat release rate (T_pHRR_), are listed in Table 4.

**Figure 5:**
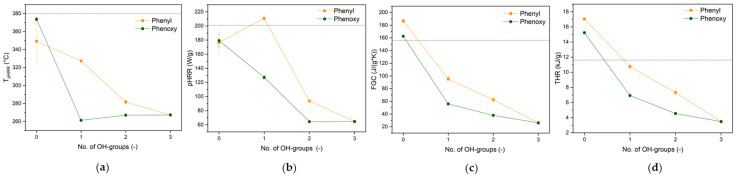
T_pHRR_ (**a**), pHRR (**b**), FGC (**c**), and THR (**d**) as a function of OH groups. The dashed horizontal lines resemble the values of pristine cotton as a reference.

Except for TPPOX and TPP, a reduction in the total heat released (THR) and the fire growth capacity (FGC) is observed, suggesting lower emissions of combustible species (Table 4). The observed trend is comparable to the one observed in TGA, as these parameters decrease with an increasing number of OH groups, with the lowest values being observed for phosphoric acid (Figure 5). This is attributed to an increasing dehydration activity and the emission of less combustible species. Similarly, phenoxy groups show enhanced efficacy over phenyl groups, as a result of the superior condensed-phase action. A reduction in the peak heat release rate (pHRR) is also observed for all species except DPPA, which is due to the fact that the flame retardant and the substrate degrade at the same time, with high caloric values.

Both TPP and TPPOX show an additional peak prior to cotton degradation, attributed to the evaporation and combustion of the FR. A significant increase in the THR over the cotton substrate (17.0 and 15.2 kJ/g, respectively) suggests that both materials act as fuel rather than FRs, as they do not interact with the substrate and combust with high energy. When one OH group is introduced, the THR is significantly reduced for phenoxy to 6.9 kJ/g, while only a negligible decrease to 10.8 kJ/g was observed for phenyl. Comparably to TGA, the phenyl system initiates cotton degradation at lower temperatures, but unlike the phenoxy system, it does not seem to influence the degradation mechanism. A similar trend was observed for the derivatives with two OH groups, as the THR of the phenyl derivative (7.3 kJ/g) is 63% higher than for the phenoxy system (4.5 kJ/g). Furthermore, the phenyl system introduces an additional signal at ~450 °C, following cotton degradation. This signal could be attributed to the pyrolysis of the P-C-bond and the concurrent emission of aromatic species.

In the TGA, it was seen that the H_3_PO_4_ sample was degrading earlier than the other derivatives, showing the smallest T_5%_ due to it exhibiting the greatest dehydration activity. The dehydration of cotton leads to the release of water, which is non-combustible and will only be detected using TGA but not MCC, as it does not contribute to heat release. However, in the case of phenoxy, the dehydration and hydrolysis of phenoxy groups occur simultaneously, leading to the emission of phenol, as was also later confirmed using TG-IR. This is evident as an additional peak preceding the substrates degradation peak, which is solely observed for phenoxy systems. This is especially obvious for the system with two OH groups, as a distinct peak at 200 °C is shown, followed by the main degradation peak, which is indistinguishable to the one observed for H_3_PO_4_, indicating that their FR mechanism is identical. The difference in the THR of ~1 kJ/g can thus be attributed to released and combusted phenol. The FGC indicates that the FR efficacy of phenoxy is increased over phenyl, with phenoxy with a single OH group even showing better performance than the phenyl derivative with two. This highlights that the apparent difference in mechanism has a huge impact on the FR efficacy.

### 3.4. Flame Test and Condensed-Phase Analysis

**Figure 6:**
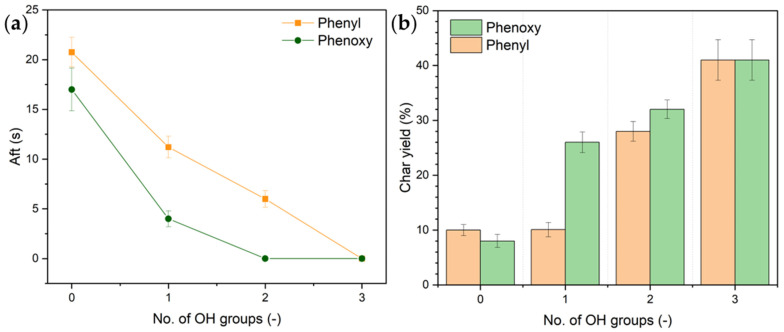
After flame time (Aft.) as a function of the number of OH groups (**a**) and the char yield after flame test (**b**). Pristine cotton shows an Aft. of 25 s.

The flame retardant efficacy was assessed using vertical flame tests according to DIN EN ISO 15025:2016. The after flame time (Aft.) was recorded and plotted as a function of the number of OH groups in Figure 6a, while the char yield, calculated from weighted circular cut-outs before and after burning, is presented in Figure 6b.

Pristine cotton exhibits an Aft. of 25 s and is fully consumed by the burning process. The application of any of the used FR species increases the FR efficacy, while solely phosphoric acid and the phenoxy derivative with two OH groups pass the flame test. However, the effect of OH group substitution differs between phenyl and phenoxy compounds. Phenyl-based derivatives exhibit a linear decrease in Aft. with increasing OH content, while an asymptotic Aft. trend is observed for the phenoxy system, suggesting a higher impact of OH groups on their flame retardant performance. This cannot be explained by stronger gas-phase activity, as P-O-bonds (360 kJ/mol) are thermodynamically more stable than P-C bonds (272 kJ/mol) and harder to pyrolyze [44]. Instead, this behavior is likely due to the hydrolysis of phenoxy groups, which facilitates phosphorus retention, as seen in the MCC results, and leads to higher char yields found after flame test, as seen in Figure 6b. A charred textile specimen after the flame test application can be found in the ESI (Figure S1).

Another potential factor for the lower efficacy of phenyl compounds is the steric hindrance of the bulky phenyl group. For instance, introducing one OH group to the phenyl system does not lead to an enhancement in charring over TPPOX (no OH groups), as the two phenyl groups disturb the interaction between the acid and the cellulosic substrate. For the corresponding phenoxy system, however, there is a significant increase in the char yield observable over the derivative with no OH groups. This might be due to the fact that phenoxy groups are not planar and can rotate away from the surface. And secondly, hydrogen bonds between the cellulose and the phenoxy–oxygen might decrease the activation barrier for acidic cotton dehydration. Once two OH groups are introduced, the obtained residues between both systems are comparable, as the OH groups can interact with the substrate unimpeded.

**Figure 7:**
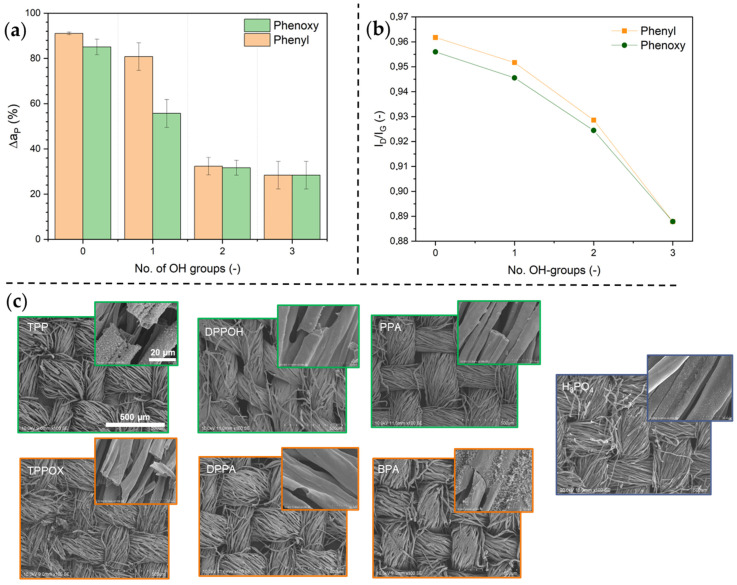
Percentual loss of phosphorus before and after flame test (**a**) and the graphitization degree of carbonaceous residues, determined using Raman spectroscopy (**b**). SEM images of residual char of phenyl-, phenoxy-, and phosphoric acid-based samples are depicted below (**c**). The scales are identical for all images.

To determine the activity of phosphorus in the condensed phase, the change in the area density of phosphorus before and after the flame test treatment (Δa_P_), calculated from ICP-OES data, is shown in Figure 7a, while SEM images of residual char are depicted in Figure 7c. Compounds with no OH groups show weak condensed-phase action, as they easily volatilize and act in the gas phase for >85%, indicated by Δa_P_. Introducing an increasing number of OH groups leads expectedly to a significant decrease in phosphorus emissions, with the H_3_PO_4_ coating transferring solely ~28% of phosphorus to the gas phase. Both the phenyl and phenoxy derivative with two OH groups are comparable to that of phosphoric acid (three OH groups) (Figure 7a). This suggests that beyond two OH groups, no significant additional phosphorus retention occurs in both systems. However, for the phenyl derivative, this trend differs from the one observed at lower OH substitution levels (one OH group), where its phosphorus retention was lower than that of the phenoxy derivative, likely due to prior mentioned steric hindrance from the bulky phenyl groups, which disrupt the interactions between the flame retardant and the cellulose substrate.

The SEM images (Figure 7c) reveal that the char structure differs significantly among the samples. For phenyl- and phenoxy-based compounds with no OH groups, the charred residues appear highly porous, and the fiber integrity is mostly lost, as evidenced by the damaged and partially fragmented fiber structures. The introduction of OH groups led to a progressive enhancement in condensed-phase activity, as reflected by the increasing residue (Figure 6b) and the reduced phosphorus loss (Figure 7a). This is further supported by the SEM images, which show that as the OH content increases, the fiber structure remains more intact, with fewer visible deformations and reduced fiber breakage.

A notable difference is observed between phenyl- and phenoxy-based compounds. Phenyl-based residues exhibit greater fiber shrinkage and detachment, indicating weaker structural reinforcement, whereas phenoxy-based residues form a more compact and interconnected structure, resembling a “glue-like” fiber morphology as the number of OH groups increase. The higher structural integrity observed in phenoxy-treated fabrics suggests an increased capacity for condensed-phase action, supporting the improved phosphorus retention seen in Figure 7a.

Raman spectroscopy (Figure S5) was employed to determine the graphitization degree and the protective capabilities of generated char. For this, the maximum intensity ratio of the D-band (1340 cm^−1^), representing disordered graphite, and the G-band (1556 cm^−1^) for ordered graphite (*I_D_*/*I_G_*) was calculated and is shown in Figure 7b as a function of the number of OH groups. Higher graphitization degrees for carbonaceous residues were obtained with increasing number of OH groups. Thus, increasing the number of OH groups does not only lead to the formation of more carbonaceous residues but also to more stable and denser structures.

Overall, phenoxy-based systems exhibit superior condensed-phase activity compared to phenyl-based systems, leading to a shorter Aft., higher char yield, and greater phosphorus retention. While OH substitution enhances flame retardancy in both systems, the effect is more pronounced in phenoxy derivatives. The steric hindrance of phenyl groups limits their flame retardant efficiency, whereas phenoxy groups, due to their flexibility and enhanced hydrogen bonding, promote dehydration and char stabilization. Finally, the transition from gas-phase to condensed-phase mechanisms becomes evident with increasing OH groups, particularly for phosphoric acid and highly substituted phenoxy derivatives.

### 3.5. Gas-Phase Analysis Using TG-IR

**Figure 8:**
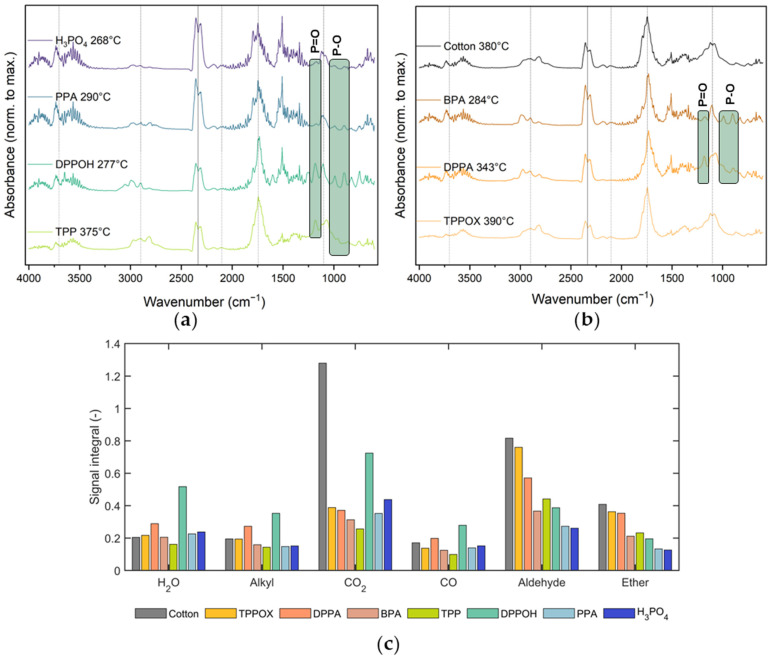
IR spectra at the maximum emission point of phenoxy (**a**) and phenyl systems (**b**). The vertical lines indicate signals originating from the cotton substrate, while the green boxes highlight PO species. Additionally, the cumulated absorbances of main cotton degradation products for all systems (**c**) are shown.

Evolved gaseous products were analyzed using TG-IR. Figure 8 shows the normalized spectra recorded at peak emission temperatures, which are analogous to TG-derived T_max_ values. Common gaseous degradation products of cotton were identified, including water vapor (3500–4000 cm^−1^) and carbonaceous compounds, such as hydrocarbons (2720–2900 cm^−1^), carbon dioxide (2337 cm^−1^, 860 cm^−1^), carbon monoxide (2103 cm^−1^, 2183 cm^−1^), carbonyls (1743 cm^−1^, 1384 cm^−1^, 1269 cm^−1^), and ethers (1100 cm^−1^) [43]. At T_max_, pristine cotton emits predominantly carbonyls, followed by ethers. Both species are primarily released via a depolymerization mechanism, while water, CO_2_, and CO are preferably emitted through the dehydration process [45]. Thus, the ratio between carbonyl and CO_2_ signals provides insights about the preferred degradation pathway.

Increasing the number of acidic OH groups enhances water and CO_2_ release while reducing the emission of volatile species (alkyls and ethers), indicating a higher dehydration activity. Phenoxy derivatives, in particular, exhibit significantly higher emissions of water at the maximum degradation temperature. However, no distinct mechanistic change is observed for TPP and TPPOX, with TPPOX even promoting lower water emissions and a higher release of a combustible species compared to pristine cotton.

To illuminate the trends of phenyl and phenoxy derivatives, cotton-derived volatiles were quantified over the entire temperature window and are depicted in Figure 8c. All FR coatings led to reduced total emissions of ethers, carbonyls, and CO_2_, evidencing flame retardant effects, even for the primarily gas-phase active species. The phenoxy derivatives, however, show significantly less cumulated emissions for combustible species (carbonyls and ethers), irrespective of the number of OH groups. This highlights the mechanistic difference between the functional groups, as phenoxy derivatives favor dehydration and suppress depolymerization, while phenyl species rather passivize degradation products in the gas phase, via radical quenching and dilution. This is further evidenced by a rapid decline in CO_2_ emissions for phenyl-based compounds, indicating that the combustion pathway towards CO_2_ is inhibited in the gas phase, but the initial formation of volatiles—and more importantly, combustibles—remains largely unaffected.

Gas-phase action was further confirmed using the identification of P = O (1192 cm^−1^) and P-O -bands (991 cm^−1^) for all FR species at the main degradation regime, indicating their role as radical scavengers and diluents. For TPPOX, P-based volatiles could be identified, as the FR is already evaporated at lower temperatures (Figure S4). As expected, the intensity of P signals decreased with higher number of OH groups, as the condensed-phase mechanism is preferred over gas-phase action, as evidenced by the Δa_P_ values (Figure 7a). As a result, P signals are almost absent for H_3_PO_4_ and phenoxy with two OH groups. Changes in the emissions of CO and H_2_O were not obvious or significant for either system. The phenoxy system with two OH groups is an exception, as CO, H_2_O, and even alkylic species were increasingly emitted, which aligns with the sharp degradation profile observed in the TGA experiment. Based on TGA and MCC, it was suspected that the P-C bond scission of phenyl species appears at 400–450 °C. TG-IR reveals bands for C-H vibrations at 3059 cm^−1^ and a sharp signal at 671 cm^−1^ at 430 °C (Figures S2 and S3). Both can be allocated towards benzene and acetylene, a known degradation product of benzene, confirming the cleavage of the P-C bond and subsequent thermal degradation [46]. This degradation mechanism was solely observable for the derivative with two OH groups, since other phenyl derivatives are not chemically bound to the cotton substrate, leading to evaporation rather than degradation.

## 4. Discussion

**Figure 9:**
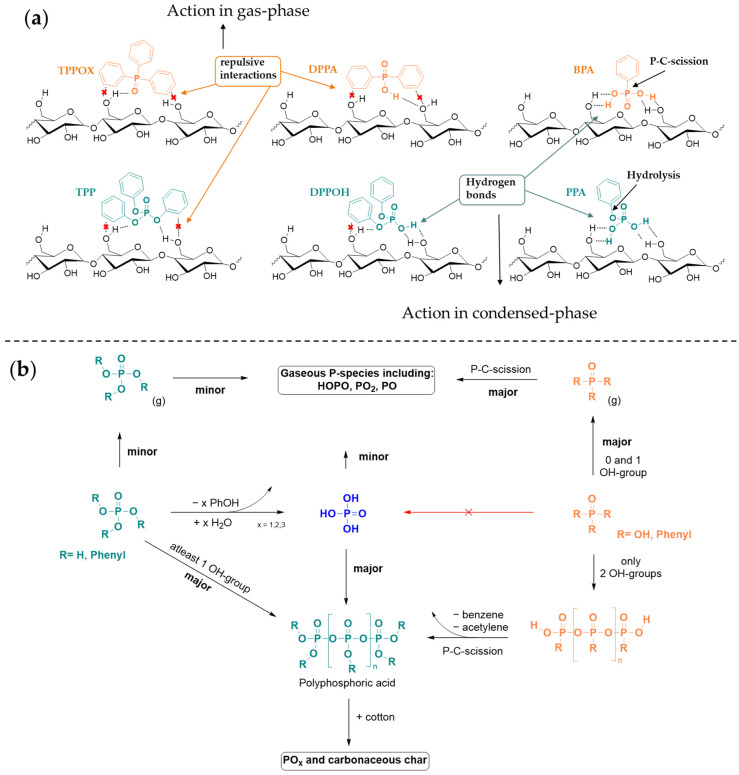
Proposed interactions between cotton, phenyl, and phenoxy FR species (**a**) and the decomposition mechanism of FR systems (**b**). Phenoxy derivatives ((**b**), **left**) show higher condensed-phase action due to hydrolyzation, while phenyl compounds degrade via P-C cleavage ((**b**), **right**).

Acidic functional groups catalyze the dehydration of cotton, favoring a degradation pathway that enhances char formation while reducing the emission of volatiles. Accordingly, the introduction of acidic OH groups shift the FR mechanism progressively towards the condensed phase and sizably increases the FR efficacy. This effect is particularly pronounced when P is functionalized with phenoxy groups rather than phenyl groups. The increased condensed-phase activity is attributed to a mechanistic shift, as the hydrolysis and dehydration activity of the phosphorus center is enhanced in an acidic environment. In contrast, phenyl groups are not susceptible to hydrolysis and do not exhibit FR activity in the condensed phase (Figure 9). Moreover, the interaction between the phosphoric acid moiety and cotton substrate is disturbed by the sterically demanding and hydrophobic phenyl groups, which is especially prominent for two phenyl groups (Figure 9a). As a result, the compound predominantly volatilizes or undergoes pyrolysis, contributing to gas-phase activity rather than condensed-phase char formation.

The efficacy of non-condensed-phase active derivatives, like TPP and TPPOX, which find use as FRs in epoxy thermosets and other systems, is minimal, effectively acting as fuel rather than FR agents [47,48,49]. This is expected, since gas-phase active species do not alter the degradation pathway of cotton substrate. For example, DOPO-based derivatives, which are well-known for their gas-phase activity, have demonstrated a poor FR efficacy on cotton fabric, with high after-flame times and a low limiting oxygen index (LOI < 25%), even at weight-gain levels of up to 20 wt.% [17,18,20]. The effectiveness could only be improved by employing a derivative with stronger condensed-phase action, as demonstrated by Ali et al. [17].

Gas-phase mechanisms focus on the passivation of degradation products, but do not interfere in their initial formation, at least for a cotton substrate. This approach is largely ineffective as cellulosic substrates inherently possess reactive pathways that can be exploited to achieve flame retardancy. Furthermore, it was seen that the scission of the P-C bond occurs at temperatures exceeding 400 °C, while the substrate degrades at 330 °C, presenting a mismatch between FR decomposition and substrate degradation. This disparity underscores the challenges associated with gas-phase active FRs. While cotton degradation shifts towards lower temperatures due to dehydration, the formation of radical species requires high activation energies and elevated temperatures, significantly reducing their effectiveness.

That being said, we cannot entirely dismiss the concept of gas-phase active FR species for cotton, given that even H_3_PO_4_ portrays minor gas-phase activity [50]. However, designing flame retardants that primarily target gas-phase activity for cotton substrates appears both impractical and relatively ineffective in enhancing the overall FR performance.

## 5. Summary and Conclusions

In this work, a comprehensive analysis was conducted to elucidate the condensed and gas-phase activity of phenyl- and phenoxy-based phosphoric acid flame retardant species on cotton fabric. Generally, phenoxy-based systems demonstrated stronger condensed-phase action and higher FR efficacies over phenyl-based systems, as assessed using standardized flammability tests. The substitution of phenoxy and phenyl groups with hydroxyl groups was found to further enhance the flame retardant performance by increasing the condensed-phase activity. Phenoxy derivatives underwent hydrolysis in the initial stages of cotton dehydration, leading to the formation of polyphosphoric acid and the enrichment of phosphorus in the condensed phase, as confirmed using ICP-OES. Phenyl derivatives, on the other hand, either exhibited negligible condensed-phase activity or volatilized at temperatures exceeding the thermal degradation point of the cotton substrate, as revealed using TG-IR analysis, explaining their limited effectiveness in the gas-phase. These findings provide valuable guidance for the design and selection of flame retardant species for cotton fabrics, particularly in the development of multifunctional hybrid coatings. It should be noted that the proposed flame retardant mechanisms may change in the presence of other additives, such as anchors, adhesives, or dyes. Moreover, this study does not address how these mechanisms translate into practical durability, including wash fastness and abrasion resistance. Additionally, the potential toxicity of the FRs and their degradation products, as well as the environmental impact of volatilized species, were not evaluated and should be considered in future studies.

## Figures and Tables

**Table 1:** Individual coating procedures, weight gain, and *a_P_* values. Orange color denotes phenyl- and green phenoxy derivatives.

No. of OH Groups	c (mol/L)	Solvent	Weight Gain Coating (wt. %)	*a_P_* (g/m^2^)
0 (TPPOX/TPP)	1.08	EtOH ^[t]^	20.5 ± 3.0	4.8 ± 0.3
	0.92	EtOH ^[t]^	22.0 ± 2.6	4.0 ± 0.3
1 (DPPA/DPPOH)	0.18	EtOH ^[3p]^	6.6 ± 1.3	2.7 ± 0.3
	0.20	THF ^[3p]^	6.1 ± 1.9	3.2 ± 0.3
2 (BPA/PPA)	0.70	EtOH ^[1p]^	3.8 ± 1.3	2.7 ± 0.3
	0.57	EtOH ^[1p]^	7.2 ± 1.8	2.6 ± 0.3
3 (H_3_PO_4_)	1.02	EtOH ^[t]^	4.7 ± 2.1	2.9 ± 0.3

[t] = tray; [xp] = x times coated using pipette.

**Table 2:** Vertical flame test obtained after flame time (Aft.), char yield, and *a_P_*. Orange color denotes phenyl- and green phenoxy derivatives.

No. of OH Groups	Aft. (s)	Char Yield (wt. %)	*a_P_* (g/m^2^)
0 (TPPOX/TPP)	20.8 ± 1.5	10.9 ± 0.9	0.4 ± 0.2
	17.0 ± 2.2	8.1 ± 0.7	0.6 ± 0.2
1 (DPPA/DPPOH)	11.3 ± 1.1	9.7 ± 1.0	0.5 ± 0.2
	4.0 ± 0.8	33.3 ± 8.8	1.6 ± 0.2
2 (BPA/PPA)	5.5 ± 1.1	28.1 ± 3.6	1.8 ± 0.3
	0.0 ± 0.0	31.7 ± 1.4	1.8 ± 0.2
3 (H_3_PO_4_)	0.0 ± 0.0	40.3 ± 3.5	2.1 ± 0.2

**Table 3:** TGA-derived parameters, including T_5%_ and T_max_, and the residues at T_max_ and 700 °C. Orange color denotes phenyl- and green phenoxy derivatives.

	T_5%_ (°C)		T_max_ (Res. %) (°C)		Res._700_ (%)	
Cotton	322		380 (42.2)		10.2	
No. of OH Groups	Phenyl	Phenoxy	Phenyl	Phenoxy	Phenyl	Phenoxy
0 (TPPOX/TPP)	227	222	380 (34.4)	368 (30.4)	5.0	7.4
1 (DPPA/DPPOH)	275	230	327 (45.8)	255 (66.4)	9.1	24.4
2 (BPA/PPA)	220	204	258 (66.5)	265 (69.7)	30.0	35.5
3 (H_3_PO_4_)	190		238 (70.6)		39.8	

**Table 4:** Micro-combustion calorimetry (MCC) parameters, including total heat release (THR), peak heat release rate (pHRR), temperature at peak heat release rate (T_pHRR_), and fire growth capacity (FGC) for pristine cotton and cotton treated with phenyl and phenoxy derivatives. Orange color denotes phenyl- and green phenoxy derivatives.

	THR (kJ/g)		pHRR (W/g)		T_pHRR_ (°C)		FGC (J/(g·K))	
Cotton	11.6 ± 0.2		200.9 ± 5.6		380.3 ± 0.5		156.0 ± 0.7	
No. of OH Groups	Phenyl	Phenoxy	Phenyl	Phenoxy	Phenyl	Phenoxy	Phenyl	Phenoxy
0 (TPPOX/TPP)	17.0 ± 0.2	15.2 ± 0.1	176.6 ± 18.0	179.3 ± 9.2	349.3 ± 22.7	373.7 ± 2.4	186.5 ± 2.5	162.6 ± 1.4
1 (DPPA/DPPOH)	10.8 ± 0.3	6.9 ± 0.1	210.8 ± 3.6	127.1 ± 2.7	327.4 ± 0.8	261.4 ± 0.7	95.4 ± 2.9	56.0 ± 1.7
2 (BPA/PPA)	7.3 ± 0.4	4.5 ± 0.1	93.9 ± 0.5	64.5 ± 1.0	281.7 ± 1.7	267.1 ± 0.4	62.6 ± 4.0	37.9 ± 1.0
3 (H_3_PO_4_)	3.5 ± 0.1		64.7 ± 0.9		267.3 ± 1.3		26.1 ± 0.4	

## Data Availability

Reference IR spectra of gaseous benzene and acetylene were taken from spectrabase and can be found here: https://spectrabase.com/spectrum/1kPEhwDaw6W, https://spectrabase.com/spectrum/3p6KRhhtRdC, accessed on 10 February 2025. The original contributions presented in this study are included in the article/Supplementary Material. Further inquiries can be directed to the corresponding author.

## Supplementary Materials

The following supporting information can be downloaded at: https://www.mdpi.com/article/10.3390/polym17070924/s1, Figure S1: Charred textile specimen after flame test application.; Figure S2: IR-spectra if evolved gases for plain BPA at 360 and 430 °C and BPA on cotton at 430 °C. Boxes highlight signals associated with acetylene/benzene and P=O; Figure S3: IR-spectra if evolved gases for BPA on cotton at different temperatures. Boxes highlight signals associated with acetylene/benzene; Figure S4: IR spectra of evolved gases at the initial degradation regime of TPP and TPPOX finished cotton; Figure S5: Raman spectra of phenoxy (left) and phenyl (right) derived char.
