# Supplementary material for: The Gas- and Condensed-Phase Efficacy of Functionalized Phosphorus Flame Retardants for Cotton Fabric: Phenyl vs. Phenoxy Groups
Source: Polymers (Basel). 2025 Mar 28;17(7):924. doi: 10.3390/polym17070924 (PMC11990989; doi:10.3390/polym17070924)
Supplement: Supplementary file 1 [file polymers-17-00924-s001.zip › polymers-3561898-supplementary.pdf]

The Gas - and Condensed-Phase Efficacy of Functionalized Phosphorus Flame Retardants for Cotton Fabric: Phenyl vs. Phenoxy Groups

Raphael Otto [1,2]*, Ava Cardona [1], Alexander M. Preußner [1], Wael Ali [1,2], Jochen S. Gutmann [1,2] and Thomas Mayer-Gall [1,2]*

[1] Institute of Physical Chemistry and Center for Nanointegration (CENIDE), University of Duisburg-Essen, Universitätsstraße 2, 45117 Essen, Germany

[2] Deutsches Textilforschungszentrum Nord-West gGmbH, Adlerstr. 1, 47798 Krefeld, Germany

* Correspondence: otto@dtnw.de (R.O.); mayer-gall@dtnw.de (T.M.-G.)

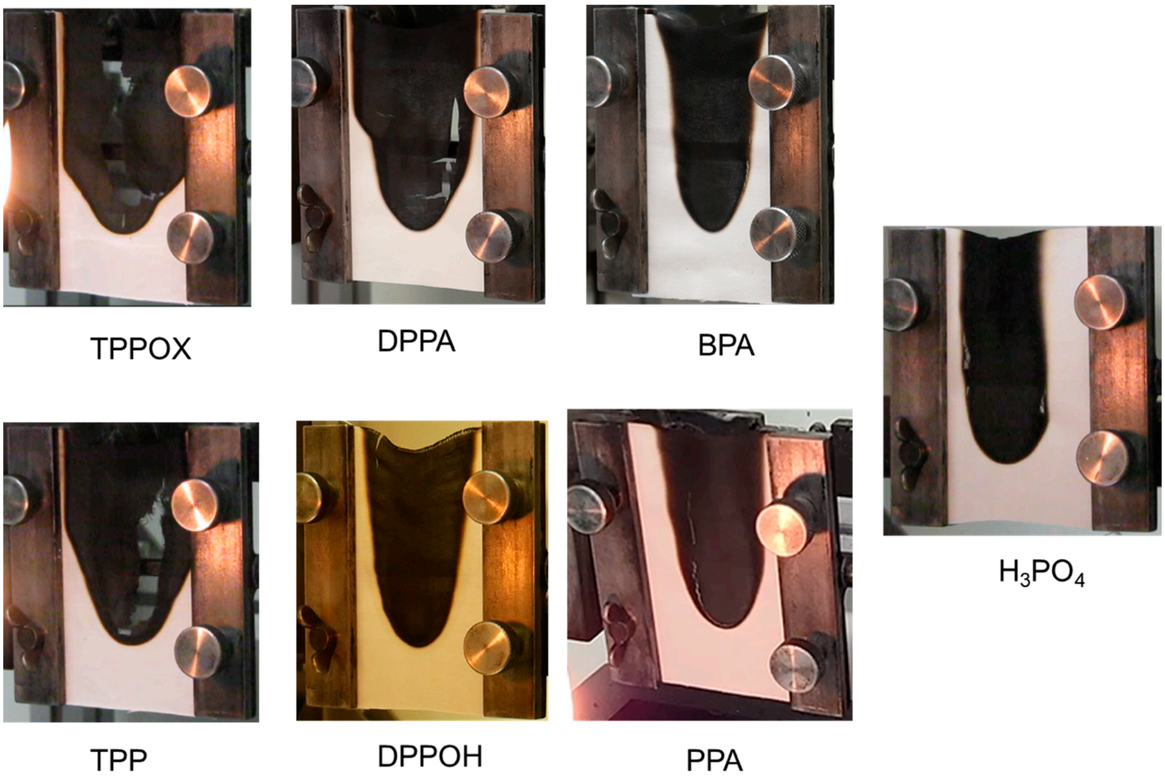

Figure S1: Charred textile specimen after flame test application.

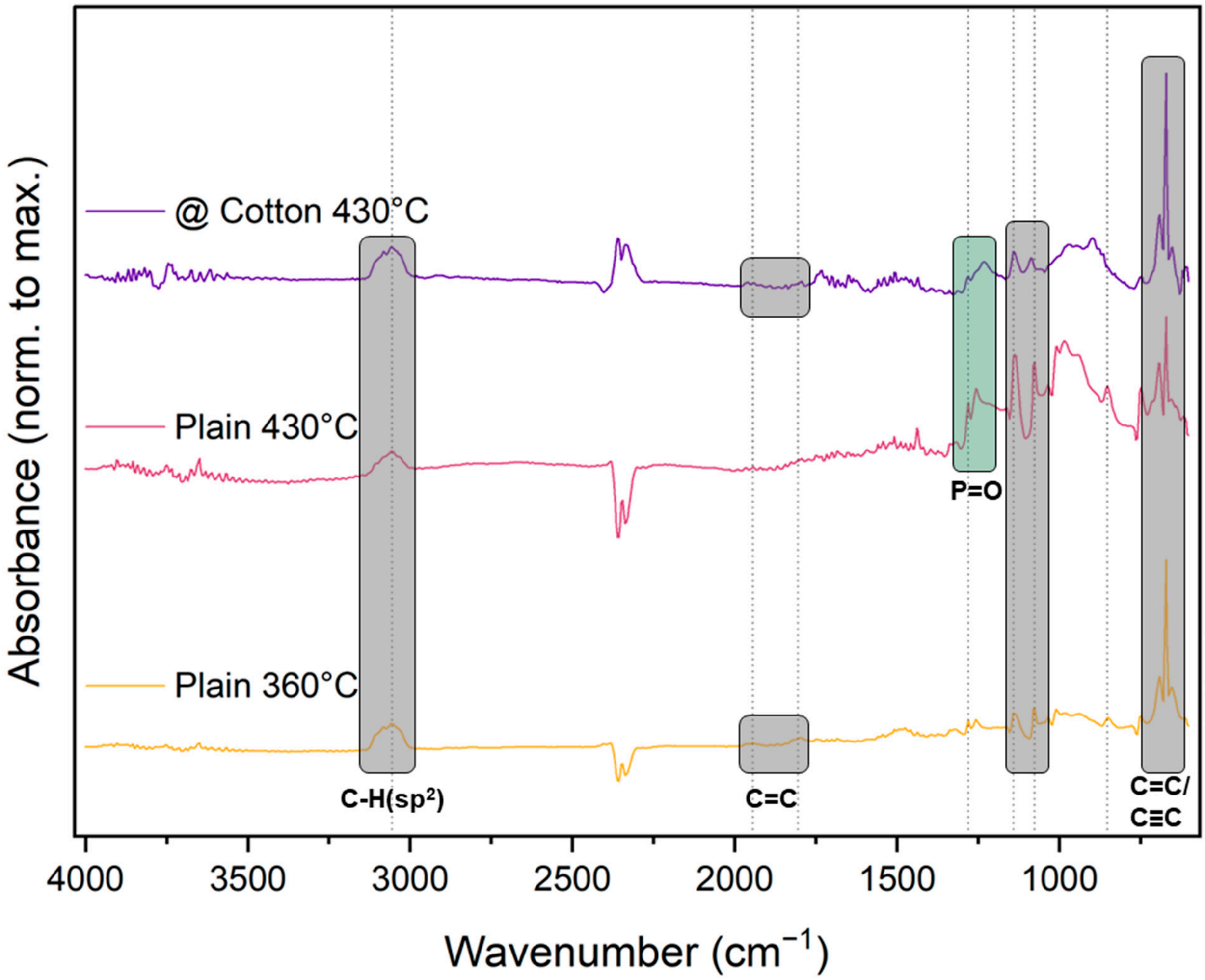

Figure S2: IR-spectra if evolved gases for plain BPA at 360 and 430°C and BPA on cotton at 430°C. Boxes highlight signals associated with acetylene/benzene and P=O.

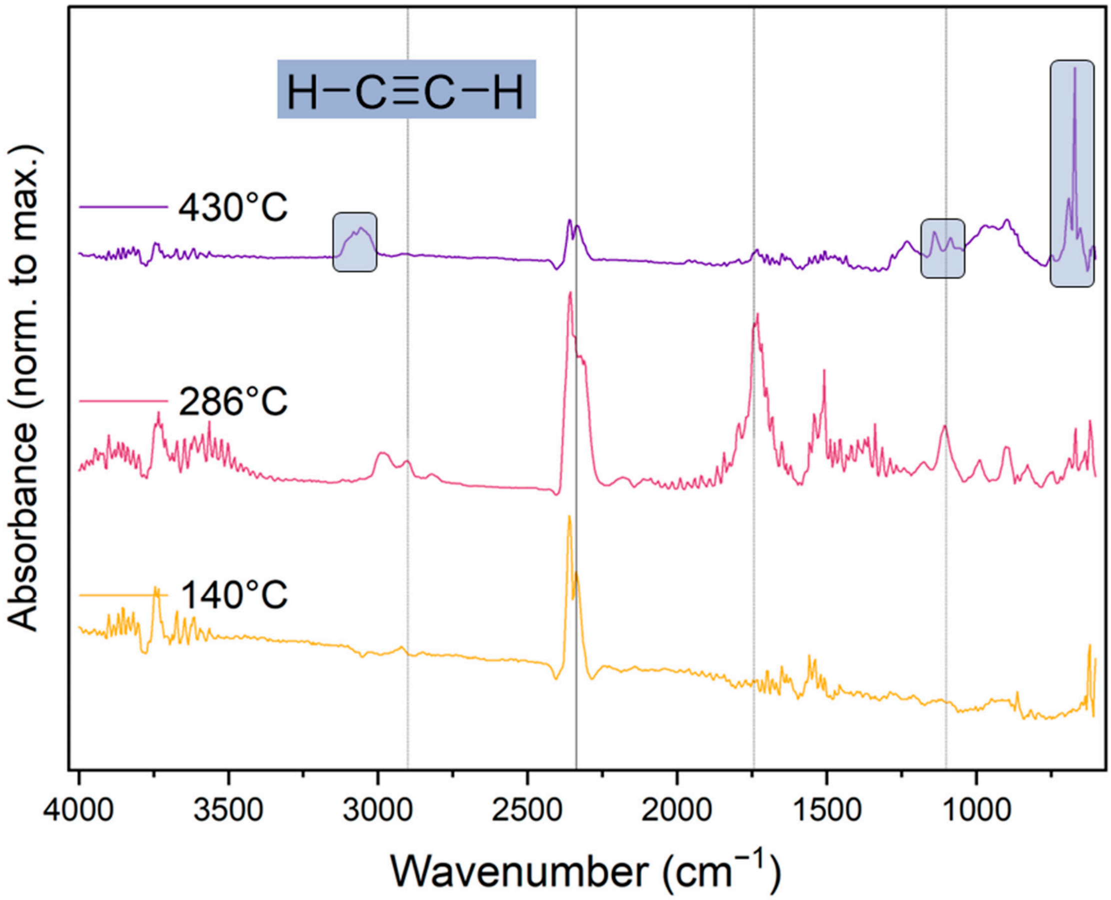

Figure S3: IR-spectra if evolved gases for BPA on cotton at different temperatures. Boxes highlight signals associated with acetylene/benzene.

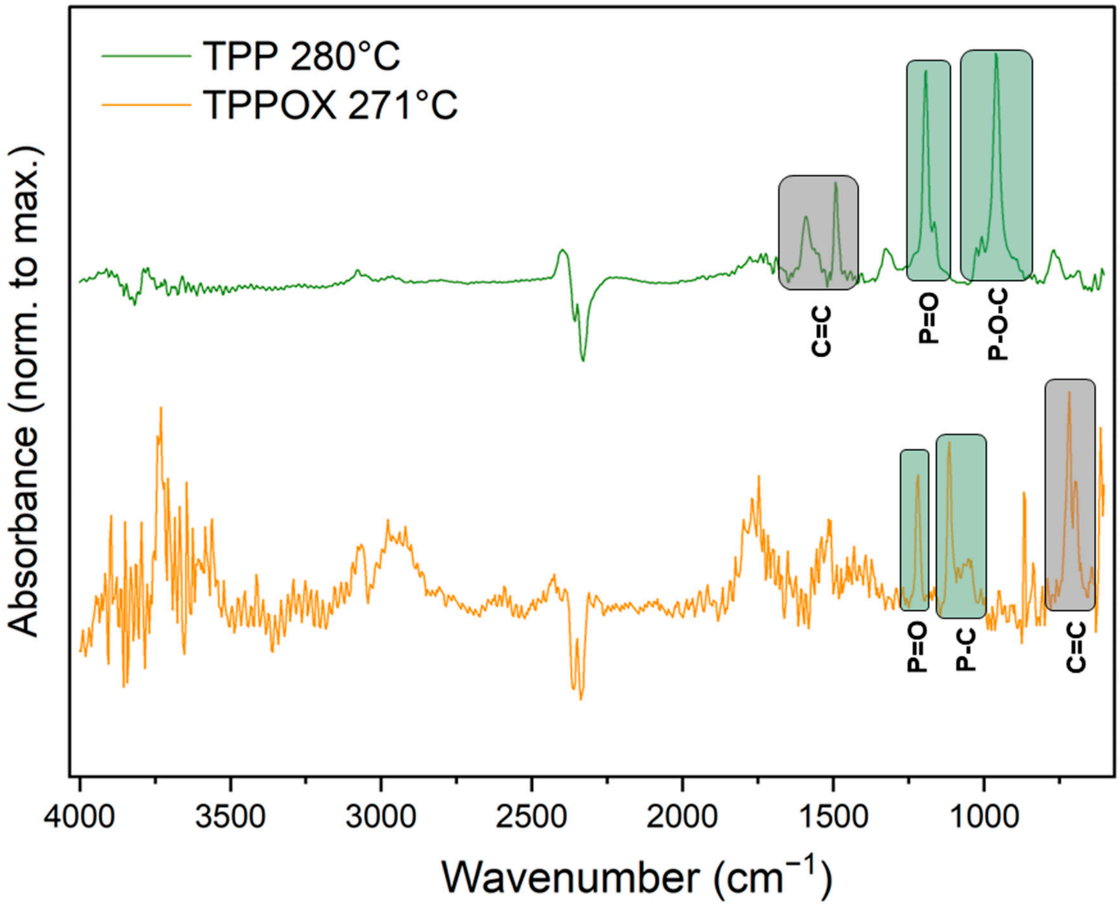

Figure S4: IR spectra of evolved gases at the initial degradation regime of TPP and TPPOX finished cotton.

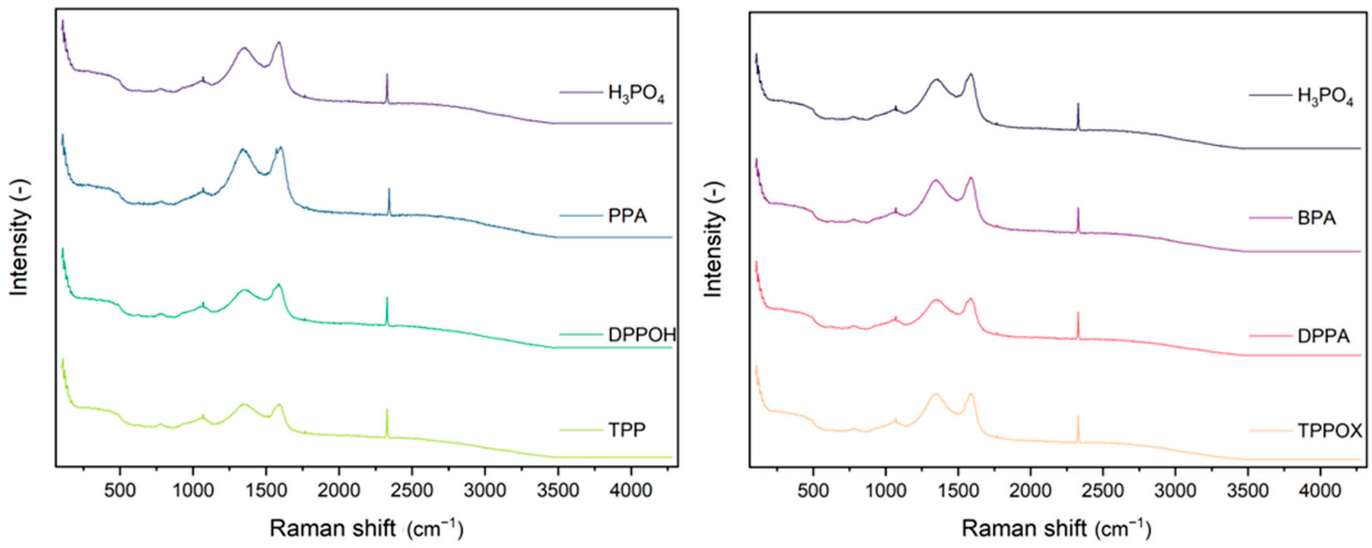

Figure S5: Raman spectra of phenoxy (left) and phenyl (right) derived char.
